# Inflammasome activation by Gram-positive bacteria: Mechanisms of activation and regulation

**DOI:** 10.3389/fimmu.2023.1075834

**Published:** 2023-01-24

**Authors:** A. Marijke Keestra-Gounder, Prescilla Emy Nagao

**Affiliations:** ^1^ Department of Immunology and Microbiology, University of Colorado Anschutz Medical Campus, Aurora, CO, United States; ^2^ Laboratory of Molecular Biology and Physiology of Streptococci, Institute of Biology Roberto Alcantara Gomes, Rio de Janeiro State University (UERJ), Rio de Janeiro, Brazil

**Keywords:** inflammasome, Gram-positive bacteria, mechanisms of activation, autophagy, regulation

## Abstract

The inflammasomes are intracellular multimeric protein complexes consisting of an innate immune sensor, the adapter protein ASC and the inflammatory caspases-1 and/or -11 and are important for the host defense against pathogens. Activaton of the receptor leads to formation of the inflammasomes and subsequent processing and activation of caspase-1 that cleaves the proinflammatory cytokines IL-1β and IL-18. Active caspase-1, and in some instances caspase-11, cleaves gasdermin D that translocates to the cell membrane where it forms pores resulting in the cell death program called pyroptosis. Inflammasomes can detect a range of microbial ligands through direct interaction or indirectly through diverse cellular processes including changes in ion fluxes, production of reactive oxygen species and disruption of various host cell functions. In this review, we will focus on the NLRP3, NLRP6, NLRC4 and AIM2 inflammasomes and how they are activated and regulated during infections with Gram-positive bacteria, including *Staphylococcus* spp., *Streptococcus* spp. and *Listeria monocytogenes*.

## 1 Introduction

Advances in cell microbiology in relation to invasive bacterial infections, signaling molecules, receptors and adhesins on target cells, and cell death mediated by bacterial virulence factors have improved our knowledge of microbial pathogenesis. Pathogen-associated molecular patterns (PAMPs) such as bacterial lipopolysaccharide, flagellin/pili or lipoteichoic acid from pathogenic microorganisms, such as *Staphylococcus* spp., *Streptococcus* spp., *Listeria monocytogenes* are responsible for activating the innate immune system (1–3). However, responses to infection by indirect detection of pathogenic molecules secreted by microorganisms and the action of proteins from the superfamily of leucine-rich repeats of the nucleotide-binding domain (NLR), in order to interrupt the signaling pathways of the host immunity, demonstrated that effector-triggered immunity promoted disruption of host cell signaling. As innocuous microorganisms do not provide pathogenic molecules to host, immunity triggered by effectors is particular for pathogenic organisms (4, 5).

Inflammasomes are signaling protein complexes that induce immune responses with the activation of inflammatory caspases, and consequent pro-inflammatory cell death called pyroptosis (6). Inflammasome activation plays a crucial role in protecting against bacterial infections organizing an effective innate immune response by controlling bacterial load and modulating the nature and magnitude of the adaptive immune response (7). Dysregulated inflammasome activation can result in an exaggerated innate immune response with tissue damage in the host. The recognition of PAMPs or microbe-associated molecular patterns (MAMPs) by phagocytes promotes the activation of inflammasomes - NLRP1, NLRP3, NLRP6, NLRC4 and AIM2 - with recruitment of adapter ASC for activation of caspase-1, secretion of interleukins pro-inflammatory such as IL-1β and IL-18, and consequent induction of pyroptosis (8). Pyroptosis, a form of cell death in immune cells, can also be activated in non-immune cells, such as hepatocytes, epithelial cells, endothelial cells, and keratinocytes (9, 10). Therefore, studies on inflammasome functions and biological responses remain of interest in improving treatments and/or prevention of inflammatory and infectious diseases. Currently, important virulence mechanisms used to avoid detection of inflammasomes and the functional consequences in host cells to evade pyroptosis induction have been studied in different Gram-positive pathogens. Therefore, this overview will focus on inflammasome activation and regulation by Gram-positive bacteria.

## 2 Inflammasomes

**Figure 1:**
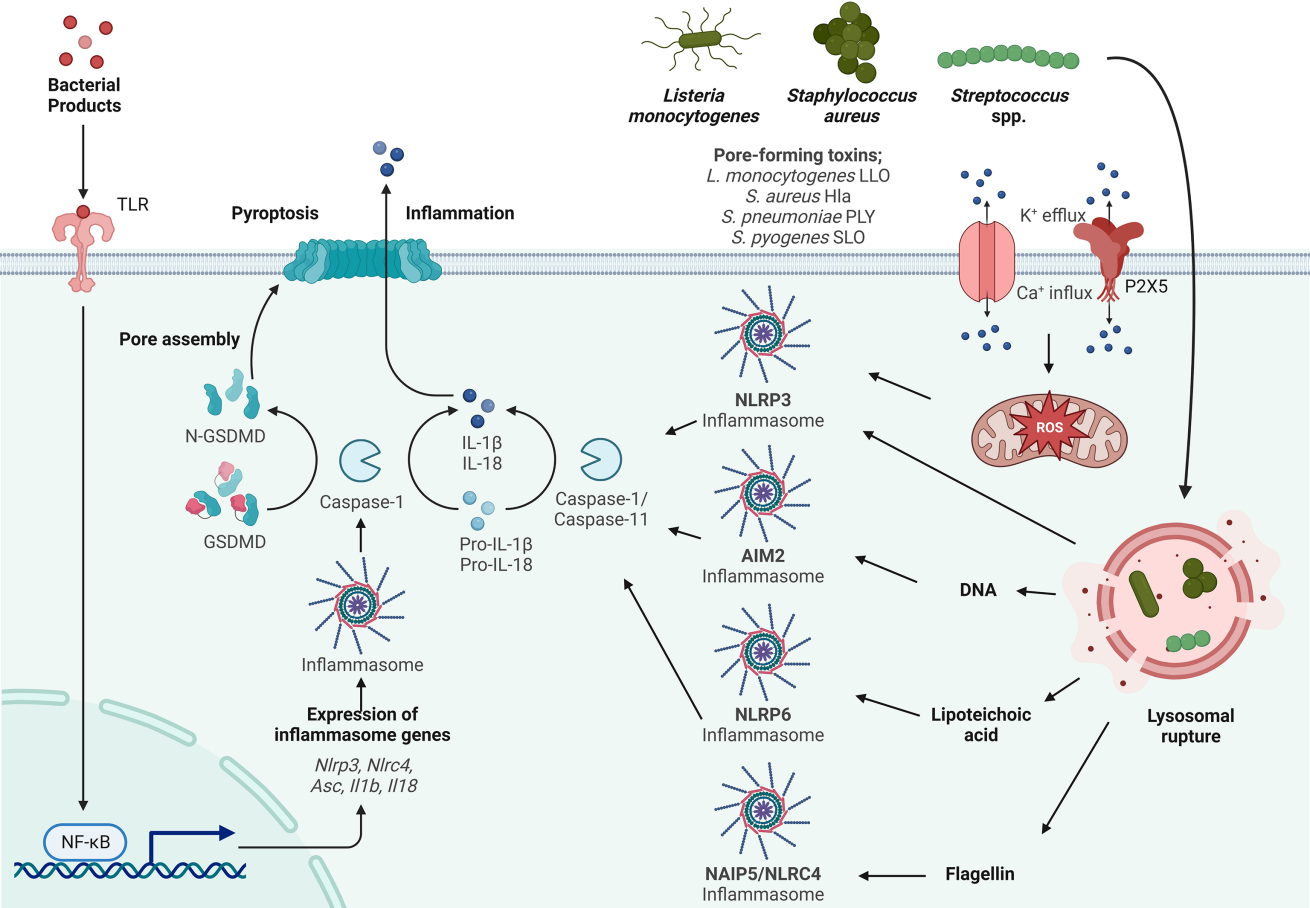
Inflammasome activation by Gram-positive bacteria. Activation of the inflammasome typically starts by priming (signal 1) through TLR activation resulting in the transcriptional upregulation of inflammasome components such as Asc, Nlrp3, Nlrc4, IL-1β and IL-18. Activation (signal 2) is initiated by a variety of PAMPs from *Listeria monocytogenes*, *Streptococcus* spp. and *Staphylococcus* spp., such as lipoteichoic acid (LTA), DNA and flagellin. LTA activates the NLRP6 inflammasome, DNA activates the AIM2 inflammasome and flagellin activates the NLRC4 inflammasome. *Listeria monocytogenes*, *Streptococcus* spp. and *Staphylococcus* spp. express pore forming toxins (PFTs) that are inserted into the plasma membrane to facilitate access to the host cytosol. K+ efflux and Ca+ influx through these pores and the P2X5 receptor (a member of the P2X purinergic receptor family of ligand-gated cation channels) results in activation of the NLRP3 inflammasome. In addition to PAMP activation and ion fluxes, the inflammasome is also activated by damage-associated molecular patterns (DAMPs) such as lysosomal rupture and production of mitochondrial reactive oxygen species (mtROS) that activates the NLRP3 inflammasome. Sensing of PAMPs and DAMPs by the NLR initiates the formation of the inflammasome consisting of ASC and Caspase-1, which in turn cleaves pro-IL-1β and pro-IL-18. Gasdermin D (GSDMD) is also cleaved and inserts into the membrane, forming pores leading to the release of the proinflammatory cytokines IL-1β and IL-18 and the induction of pyroptosis.

The innate immune system is the first barrier pathogens encounter and that will protect the host from infections. Pattern recognition receptors (PRRs) are a class of innate immune receptors that detect PAMPs (11, 12). One family of the PRRs are the nucleotide-binding and oligomerization domain (NOD)-like receptors (NLRs) (13). To date, 22 NLRs have been identified in humans and they are categorized into subfamilies based on the type of N-terminal signaling domain (13). The NLRP receptors contain a pyrin domain (PYD) and NLRC receptors contain a caspase activating recruitment domain (CARD) (14). Both NLRPs and NLRCs can form a multiprotein complex called the inflammasome that consists of the adaptor apoptosis associated speck-like protein containing a CARD (ASC) and caspase-1 (15). ASC contains a PYD and a CARD domain and binds the NLRs *via* either PYD-PYD or CARD-CARD interaction, respectively. The interaction of the NLR with ASC leads to the recruitment of procaspase-1 that subsequently is cleaved into the active caspase-1. Caspase-1 cleaves the proforms of interleukin (IL)-1β and IL-18 and gasdermin D (16–18). Cleaved gasdermin D inserts itself into the plasma membrane to form pores that mediate the release of IL-1β and IL-18 and drive the cell death program pyroptosis (19, 20). Inflammasome activation requires two steps of stimulation; signal 1 is known as ‘priming’, and signal 2 as ‘activation’ (21). Priming can be achieved by activation of Toll-like receptors (TLRs) to promote the activation of NF-κB leading to the transcriptional expression of key components of the inflammasome, such as pro-Il-1β, pro-IL-18 and the NLR itself. Signal 2 is the stimulus that activates the inflammasome (**Figure 1**). In addition to PAMPs, Damage/Danger Associated Molecular Patterns (DAMPs) which are endogenous danger signals released from damaged tissues can also trigger inflammasome activation (22). In this review, we will focus on the NLRP3, NLRP6, NLRC4 and AIM2 inflammasomes and their activation by Gram-positive bacterial pathogens.

### 2.1 The NLRP3 inflammasome

NLRP3 is expressed in innate immune cells, mainly macrophages, and NLRP3 is one of the most widely studied inflammasome-forming NLRs. The NLRP3 inflammasome is activated by an increasing number of stimulators, including viruses, fungi, bacteria, pore-forming toxins, reactive oxygen species (ROS), extracellular ATP, potassium efflux and calcium influx, mitochondrial dysfunction, nigericin, urate crystals, amyloid-B, and various environmental insults such as asbestos and silica. Direct binding of a ligand to NLRP3, however, remains unclear since all stimuli are structurally and chemically very different (23). Instead, it is believed that NLRP3 can sense a common cellular event that is induced by the wide variety of stimuli, such as potassium efflux or production of ROS, however the underlying mechanism remains to be determined. The NLRP3 inflammasome is important for maintaining a balanced in the gastrointestinal tract since *Nlrp3^-/-^* mice displayed a unique intestinal microbiota and are more susceptible to experimental colitis (24–26). *Nlrp3^-/-^* mice were also found to have reduced expression of AMPs, which could potentially influence the microbiota composition (25).

NLRP3 is also involved in the non-canonical inflammasome which is activated by the binding of cytosolic lipopolysaccharides (LPS) of Gram-negative bacteria to the murine inflammatory caspase-11 (27, 28). In humans, the orthologs of murine caspase-11 are caspase-4 and -5 (29). Cytosolic LPS induces self-cleavage and oligomerization of caspase-11 that subsequently processes gasdermin D to induce pore formation in the plasma membrane. The pore formation leads to potassium efflux, which in turn activates the NLRP3inflammasome resulting in the processing of pro-IL-1β and pro-IL-18 and secretion of IL-1β and IL-18 (30, 31). Caspase-11 is widely expressed in a variety of cells of both the hematopoietic- and non-hematopoietic lineage, including epithelial cells and macrophages (32). It was suggested that since caspase-11 directly binds to LPS it only contributes to defense against gram-negative bacteria, however, a recent study reported that caspase-11 plays a crucial role in generating immune responses against *Listeria monocytogenes* and *Staphylococcus aureus* infection (see paragraph 2.3. The NLRP6 inflammasome) (33).

### 2.2 The NLRC4 inflammasome

The NLRC4 inflammasome is activated in response to flagellin and the needle and inner rod protein of Type III and Type IV secretion systems (T3SS, T4SS) of Gram-negative bacteria, such as *Salmonella* Typhimurium, *Shigella flexneri*, *Legionella pneumophila* and *Pseudomonas aeruginosa*. These ligands do not directly bind to NLRC4, but instead, NLRC4 requires the NLR family apoptosis inhibitory proteins (NAIPs) to sense and bind to the bacterial ligands. Murine NAIP1 binds the needle protein, NAIP2 the inner rod protein and NAIP5/6 binds flagellin (34–36). Humans have two isoforms, NAIP and the extended NAIP*. NAIP* binds to flagellin, whereas NAIP binds the needle and rod proteins (37–39). Direct binding of flagellin to NAIP5 changes the conformation of NAIP5 allowing recruitment and binding of NLRC4, which promotes further recruitment and activation of the next incoming NLRC4 protein, resulting in a wheel-shaped structure consisting of a single flagellin-NAIP5 and NLRC4 protomers (40–44). Upon completion of the formation of the wheel-shaped structure, the CARDs of NLRC4 interact and recruit ASC molecules to initiate ASC polymerization into long filaments, known as the ASC specks. The CARD of ASC is exposed on the outside as serves as a docking station for pro-caspase-1 (45). The NLRC4 inflammasome can also function without ASC, since macrophages deficient of ASC display similar levels of pyroptosis in response to NLRC4 ligands (46, 47). The gram-positive bacteria *Staphylococcus aureus* and *Listeria monocytogenes* have also been demonstrated to activate the NLRC4 inflammasome. The specific ligand and the exact underlying mechanism how *S. aureus* (non-flagellated, no T3SS or T4SS) can activate the NLRC4 inflammasome is currently unknown (48). *L. monocytogenes* flagellin activates the NLRC4 inflammasome (49, 50).

### 2.3 The NLRP6 inflammasome

NLPR6 is a lesser studied NLR and is predominantly expressed in the intestine. Similar to the NLRP3 and NLRC4 inflammasome, NLRP6 assembles into a multiprotein complex consisting of ASC and caspase-1, promoting cleavage and secretion of inflammatory cytokines and cleavage of gasdermin D to initiate pyroptosis (51). One of the first reports on NLRP6 demonstrated that it is important in maintaining homeostasis of the microbiome (52). Changes in the composition of the microbiome results in an imbalance in the microbial community that is associated with a wide range of human diseases, such as inflammatory bowel disease, cancer, obesity, and diabetes. Dysbiosis can be established in multiple different ways, for example during infections, caused by mutation in host factors or changes to the diet. Additionally, the host immune system has also been demonstrated to be important in maintaining homeostasis of the microbiome. NLRP6 activation was one of the first host factors of the innate immune system suggested to regulate the microbiome composition (52). It was demonstrated that *Asc^-/-^*, *Caspase-1^-/-^*, *Nlrp6^-/-^* and *Il18^-/-^* mice, but not *Il1b^-/-^ or Il1R^-/-^* mice, developed severe colitis after dextran sodium sulphate (DSS) treatment and that co-housed wild type mice were also more susceptible to DSS treatment, indicating that the microbiota is responsible for the exacerbation of DSS-induced colitis (51, 52). These findings, however, were later challenged as it was demonstrated that *Nlrp6^-/-^* and *Asc^-/-^* mice and littermate control mice did not reveal any differences in the microbiome composition, nor were they more susceptible to DSS treatment (53). They argued that the differences in the microbiome could be associated with the facilities where the mice are housed. In the report by Levy and co-workers, it was demonstrated that the microbiota-modulated metabolite taurine can activate the NLRP6 inflammasome resulting in pro-IL-18 processing and IL-18 secretion. IL-18 mediates production of antimicrobial peptides (AMPs) and a balanced AMP landscape is required for the maintenance of intestinal homeostasis (51). In contrast, the levels of the metabolites histamine and spermine were increased in *Asc^-/-^* mice, and both histamine and spermine suppressed IL-18 production in colon explants. These data indicate that microbiota-modulated metabolites can either positively or negatively regulate the NLRP6 inflammasome and their absence/presence in the gastrointestinal tract can have major effects on gut health and disease (51). NLRP6 expression in intestinal epithelial cells protects against colorectal cancer and colitis *via* a mechanism dependent on caspase-1 mediated IL-18 production (52–55). NLRP6 has also been demonstrated to be expressed in goblet cells where it regulates mucus production (56). Interestingly, *Nlrp6^-/-^* mice are more resistant to infection with *Listeria monocytogenes*, *Salmonella* Typhimurium and *Escherichia coli*, demonstrating NLRP6 can act as a negative regulator in innate immune signaling and host defense (57). Similarly, NLRP6 serves as a negative regulator during infection with *Staphylococcus aureus* (58). In a later report it was demonstrated that in response to *Listeria monocytogenes* and *Staphylococcus aureus* infection, NLRP6 directly binds to lipoteichoic acid (LTA) in macrophages leading to the recruitment of both caspase-1 and caspase-11 resulting in IL-18 secretion (33). In addition to its role in the host defense against bacterial species, NLRP6 can also bind to RNA from enteric viruses, and it can sense microbiota-associated metabolites (51, 59). In response to viral RNA, however, the NLRP6 does not form an inflammasome with ASC and caspase-1, but instead binds the RNA helicase DHX15 and interacts with mitochondrial antiviral signaling protein (MAVS) to induce a type I interferon (T1IFN) response (59). Although NLRP6 can form the classical inflammasome, many aspects of NLRP6 are still poorly understood; NLRP6 plays a major role in maintenance of microbiota homeostasis, can bind distinct ligands, is anti-bacterial and anti-viral, but is also a negative regulator of innate immune signaling. Future studies will have to determine the underlying mechanisms of NLRP6 activation and immune signaling during infections.

### 2.4 The AIM2 inflammasome

Absent in melanoma 2 (AIM2) is a member of the HIN200 family and is a cytosolic sensor of double-stranded DNA (dsDNA). It is the first non-NLR receptor identified that can form an inflammasome with ASC and caspase-1 (60–63). Upon binding of bacterial, viral and mitochondrial dsDNA to the HIN200 domain of AIM2, multiple sensors cluster together resulting in binding of ASC *via* PYD-PYD interaction. The CARD domain of ASC subsequently recruits procaspase-1 to the complex allowing for autocleavage of caspase-1, processing of pro-IL-1β, pro-IL18 and gasdermin D, resulting in cytokine secretion and pyroptosis. Gram-positive bacteria, including but not limited to *Staphylococcus aureus* and *Listeria monocytogenes* release dsDNA in the cytosol after escaping the phagolysosome that activates the AIM2 inflammasome (64, 65).

## 3 Gram-positive bacteria trigger inflammasome activation

Host immune programs, including inflammasomes, play a crucial role in limiting the spread of pathogens and maintaining tissue function and integrity. Thus, pro-inflammatory transcription factors orchestrate the magnitude and duration of immune responses. Dysregulation of immune response can lead to uncontrolled inflammation promoting serious disease (66–68). In this way, several pathogenic microorganisms have evolved mechanisms to prevent inflammasome activation.

### 3.1 *Listeria monocytogenes* - Advantage of inflammasome activation

*L. monocytogenes* is a Gram-positive bacterium responsible for listeriosis acquired by contaminated food. Murine or human macrophages infected with *L. monocytogenes* demonstrated activation of multiple inflammasomes, including AIM2-ASC, NLRP3-caspase-1, NAIP5-NLRC4, NLRP6-caspase-11, caspase-1, caspase-11, and NLRP1B (69, 70). In addition, cytosolic listeriolysin O (LLO) and lipoteichoic acid (LTA) have also been implicated in inflammasome activation (**Table 1**).

**Table 1:** Inflammasome activation by Gram positive bacteria.

Pathogen/ virulence factor	Responsible factor	Effect	Pathway	References
*Listeria monocytogenes*, LLO, cytosolic LTA, flagellin	NLRP1B, NLRP 3, NLRP 6-caspase-11, NAIP5-NLRC4,AIM-ASC-caspase-1, IL-1β, IL-18, gasdermin D; P2X5	Exacerbation of systemic *L. monocytogenes* infection and an extent ROS production. NLRP1B detects a signal generated by metabolic stress; LLO phosphorylate ASC that exacerbates infection. P2X5 as a critical mediator of protective immunity.	NF-κB/ROS and ERK, Mst1/2-ALK-JNK, Lyn-Syk signaling	(33, 71–75)
*Staphylococcus aureus*, Pore- fo et al., 2021rming secreted toxins, lipoproteins, staphylokinase, staphylococcal enterotoxin A	NLRP1B, NLRP3, AIM-ASC-caspase-1, Caspase-1, gasdermin D	Determine the outcome of infection by controlling the bacterial load and shaping the nature and magnitude of the adaptive immunity response. AIM2 signaling pathway might be a therapeutic target to control unwelcome inflammation.	K+ efflux,NF-κB and MAPK, TREM2/β-catenin	(76–81)
*Staphylococcus epidermidis*, serine protease Esp	NLRP3, Caspase-1, IL-1β	Extracellular proteases during pathogenicity mediated processing to mature IL-1β.	?	(82)
*Streptococcus agalactiae*, pore-forming β-h/c protein, sialic acid	NLRP3-ASC, Caspase-1, IL-1β, IL-18,	Modulate the phagocytic and bactericidal responses of neutrophils and monocytes to S. agalactiae infection. Siglec- 14 enhances and Siglec-5 inhibits inflammasome activation in response to *S. agalactiae* infection.	Protein kinase D, NFκB activation, Siglec-14 signaling	(83–87)
*Streptococcus pneumoniae*, NanA, PLY	NLRP3, NLRP 6, AIM-ASC-caspase-1 Caspase 11, IL-1β, gasdermin D	Protection against *S. pneumoniae* infection. NLRP6 negatively regulates the inflammatory signaling pathway in macrophages.	K+ efflux, Akt Syk and JNK kinases,RIPK3	(88–91)
*Streptococcus pyogenes*, M1 protein, LTA, streptolysin O, streptolysin S	NALP3/NLRP3 gasdermin A	Inflammasome-dependent signaling favors inflammation in invasive *S. pyogenes* infections.	K+ efflux NF-kappaB	(92–95)
Streptococcus equi subsp. zooepidemicus	IL-1β, IL-18	Gasdermin D protects against Streptococcus equi subsp. zooepidemicus infection	miR-223-3p	(96)
*Propionibacterium acnes*, metalloprotease	NLRP3, IL-1β, IL-18,gasdermin D	Induction of inflammation in acne lesions.	K63 deubiquitination NF-κB/MAPK	(97–99)
*Corynebacterium pseudotuberculosis*	NLRP3, Caspase-1, IL-1β	Mediation of the acute phase of inflammation and an important role in the response to *C. pseudotuberculosis* infections	NF-κB, p38MAPK	(100)
*Bacillus anthracis*, LeTx	NLRP1B, NLRP3, Caspase-1, IL-1β, IL-18, gasdermin D	The activation of inflammasome is required for macrophage lysis and contributes to death	TRIF signaling, MAPKK/p38/MK2 signaling RIPK1 Kinase	(101, 102)
*Clostridium difficile*, SLP	NLRP3, Caspase-1, IL-1β, IL-18, gasdermin D	Regulating host defense against *C. difficile* infection	mTOR pathway, ATP-P2X7 pathway ATP-P2X7 pathway	(103, 104)
*Clostridium septicum*	NLRP3, Caspase-1,IL-1β, IL-18, gasdermin D	Rapid inflammasome-mediated lethality in mice	Mg^+2^ and K^+^ efflux	(105)

NLRs, NOD-like Receptors; LLO, listeriolysin O; LTA, lipoteichoic acid; TREM2, Triggering of Receptors Expressed in Myeloid Cells 2; NanA, Pneumococcal sialidase; PLY, pneumolysin; LeTx, Lethal toxin; SLP, Surface layer proteins.

After endocytosis, *L. monocytogenes* promotes lysosomal disruption, leading to the release of lysosomal contents, translocation of Listeria to the cytoplasm and inflammasome activation. NLRP3 is activated when *L. monocytogenes* escapes the vacuole in conditions with limited cytosolic replication and lysosomal damage, and NLRC4 is activated by the bacterial flagellum. However, AIM2 recognition may predominate in conditions with high cytosolic replication. AIM2-ASC-caspase-1 is the inflammasome sensor of *L. monocytogenes* DNA and its expression requires type I IFN signaling (106). AIM2 connects DNA directly, resulting in atypical inflammasome with the activation of caspase-1, IL-1β and IL-18 and consequent induction of pyroptosis (71, 107).

Gao and co-workers (72) showed that infection by *L. monocytogenes* induced the activation of the NLRP3 inflammasome through the Mst1/2-ALK pathways; as well as the participation of the interaction between Nek7 and NLRP3 *via* JNK that promoted the intensification of the host defense against Listeria infection by increasing the maturation and release of the pro-inflammatory cytokine IL-1β. Furthermore, pore-forming toxin and LLO played a critical role in the process. Recently, an increase in transcriptional levels of inflammatory factors was demonstrated, where levels of proteins associated with NLRP3, NF-κB and ERK were increased after Listeria infection and consequent inflammation of the central nervous system (73). Therefore, blocking inflammasome activation could decrease inflammation and improve patient survival and prognosis as a potential therapeutic intervention target for *L. monocytogenes* infection (108).

Interestingly, mouse macrophages infected with *L. monocytogenes* activated NLRP6 and caspase-11. NLRP6, activated by Gram-positive bacteria or lipoteichoic acid (LTA) in the host cytosol, has a function to limit inflammation and to regulate systemic infection by pathogens (109, 110). Activation of NLRP6 in macrophages infected by *L. monocytogenes* or LTA induced caspase-11 processing which promoted IL-1β/IL-18 maturation, caspase-1 activation and regulation of IL-18 secretion, revealing a novel signaling pathway by which the NLRP6 inflammasome was activated by cytosolic LTA, recruiting pro-inflammatory caspases in systemic infection by Gram-positive bacterial pathogens (33).

NLRP1B is activated under conditions that lead to a decrease in cytosolic ATP (76), suggesting that energy reduction may allow NLRP1B to detect intracellular pathogens, including Gram-positive bacteria. The pore-forming toxin and LLO were required for the rupture of the vacuolar membrane, reduction of ATP levels and energy stress through disruption of mitochondrial function by *L. monocytogenes*. In addition, Listeria invasion into the cytosol may contribute to ATP depletion. Listeria-infected fibroblasts expressed NLRP1B, pro-caspase-1 and IL-1β, suggesting that NLRP1B detected a signal generated by metabolic stress (74). Another study showed that LLO was recruited and oligomerized leading to activation of Lyn and Syk kinases in lipid membrane rafts. Upregulation of ASC phosphorylation was verified in a secondary step, showing that ASC phosphorylation can utilize another signaling pathway in addition to LLO-mediated Lyn-Syk kinases. These data demonstrated that pathogens could activate different inflammasomes in order to control immune responses and benefit the spread of the pathogen in the host (75).

During inflammasome activation, the increased influx of macrophages may be detrimental to the defense against *L. monocytogenes in vivo*. The Mint3-mediated pathway, which regulates ATP production in macrophages, has contributed to the regulation of severe listeriosis in mice, suggesting a potential therapeutic target (111). In this way, the regulation of gasdermin D by caspase-1 was enhanced in Mint3–/– mice infected with Listeria, accompanied by a strong induction of pyroptosis and bacterial shedding (112). Other molecules have also been described as activating inflammasomes; among them the P2X7 receptor, a potent activator of NLRP3 inflammasome and the release of the pro-inflammatory cytokines IL-1β and IL-18 (113). However, very little is known about P2X5, belong to the P2X family, in the immune system. Recently, P2X5 has been shown to play an important role in protective immune responses associated with inflammasome activity, including *in vivo* infection by *L. monocytogenes* (114).

The protein kinase, p38-regulated/activated protein kinase (PRAK), contributes to the regulation of cell proliferation, stress responses and cell migration. Furthermore, PRAK dysfunction impaired the antibacterial activity of neutrophils and the formation of neutrophil extracellular traps (NETs) (115). Assays with PRAK-deficient mice showed high mortality when compared to wild-type mice after infection by *L. monocytogenes*. In addition, Listeria-induced autophagic activities were reduced in the absence of PRAK. In this way, PRAK potentiated bactericidal activity with increased ROS production, inflammasome activation and autophagy induction, providing a new perspective for innate immunity against listeriosis (116).

### 3.2 Staphylococcus spp

*Staphylococcus aureus* is responsible for human infections such as pneumonia, endocarditis and sepsis with high morbidity/mortality and consequent increase in hospital costs (117). *S. aureus* can manipulate cell death by apoptosis, necrosis or pyroptosis to colonize and establish infection in different hosts (118). The inflammasome plays a crucial role in rising an effective immune response against *S. aureus* infection, controlling bacterial load and the magnitude of the adaptive immune response (7). *S. aureus* pore-forming toxins (α-toxin, LukAB and PVL) induced NLRP3-ASC inflammasome activation in human and murine phagocytes *in vitro*. In addition, methicillin-resistant *S. aureus* strain activated NLRP3-ASC inflammasomes through K+ efflux, caspase-1 activation, and release of IL-1β and IL-18 cytokines with induction of pyroptosis (77, 78). Peptidoglycan resistant to lysozyme strongly inhibited IL-1β production in response to infection *in vitro* and *in vivo*, suggesting that the *S. aureus* subverts IL-1β secretion by modifying its peptidoglycan from cell wall (119). Thus, new therapeutic approaches targeting peptidoglycan degradation in host immune cells could control bacterial infections.

ASC assembly and AIM2 activation by intracellular *S. aureus* after suppression of perforin-2 were described for the first time by Pastar and co-workers (79). In this work, *S. aureus*-infected diabetic foot ulcers triggered the AIM2 inflammasome activation resulting in activation of gasdermin D, and increased levels of IL-1β in tissue samples from chronic ulcers in diabetic patients, contributing to prolonged inflammation. Additionally, phagocytosis and bacterial degradation were related to the activation of NLRP3 inflammasomes and IL-1β secretion in response to both live *S. aureus* and *S. aureus* peptidoglycan. Interestingly, IL-1β production was mediated by the NLRP3 activation, and the secretion of IL-6, KC and CCL2 was AIM2-dependent (120). Together, the NLRP3/AIM2 inflammasomes played a critical role in *S. aureus* infection and could be a therapeutic target to control unwanted inflammation.

The involvement of extracellular vesicles serving as a secretory pathway for the transport of protected bacterial cargo (virulence factors, surface glycoproteins, proteases, etc) to host cells have been reported in several Gram-positive bacteria. Activation of NLRP3 by *S. aureus* extracellular vesicles have been shown to be dependent on the load of pore-forming toxins and K+ efflux. The critical role of pore-forming toxins was associated with *S. aureus* extracellular vesicles in triggering inflammasome activation, modulating of innate host immune response during *S. aureus* infection. In this way, extracellular vesicles serve as an important secretion system that allows the transport of molecules to host cells during infections to modulate cellular functions (121). Similarly, after endocytosis of *S. aureus* by bovine mammary epithelial cells, the bacteria escaped from the endosomal vesicles, causing inflammation and cell death through the K+ efflux and NLRP3 activation, and consequently pyroptosis of MAC-T cells (80).

Staphylococcal enterotoxin A promotes intestinal barrier dysfunction, triggering an inflammatory response with activation of the NLRP3 inflammasome and the NF-κB/MAPK signaling pathways. Selective inhibitors of NF-κB/MAPK pathways in THP-1 cells attenuated the expression of inflammasome-associated proteins that were upregulated by staphylococcal enterotoxin A. Thus, the development of compounds acting on enterotoxin A may be a promising strategy for the prevention and treatment of diseases caused by the toxin (81). Moreover, the triggering of receptors expressed in myeloid cells 2 (TREM2) is considered a protective factor for the host against bacterial infection; however, their role is unclear. TREM2 or its adapter molecule DAP12 activates β-catenin, the molecule responsible for maintaining cell viability and preventing cell death, in myeloid cells stimulated by M-CSF. Study showed that TREM2 inhibited macrophage pyroptosis induced by *S. aureus* and *Streptococcus pneumoniae*, providing a new host defense mechanism against pathogenic microorganisms (122–124). In addition, TREM2 promoted the stabilization and phosphorylation of β-catenin in the nucleus and cytoplasm and down-regulation of NLRP3 inflammasome activation. TREM2/β-catenin also inhibited ASC oligomerization and ASC-NLRP3 association with suppressed pyroptosis in macrophages infected by pyogenic bacteria (80).

Potential role of staphylokinase in the virulence of community associated *S. aureus* remains unknown. Furthermore, a significant role of staphylokinase in the acute pneumonia model involved the activation of innate immune signals, especially pathways related to the NLRP3 inflammasome. The investigations revealed that staphylokinase contributed to NLRP3 inflammasome activation, increasing K+ efflux, ROS production and NF-κB signaling, exacerbating pulmonary infection. These data improve our understanding of the mechanism of action of staphylokinase in bacterial pathogenesis (80).

Recently, researchers showed that *Staphylococcus epidermidis* strains induced caspase-1-independent IL-1β release and NLRP3 inflammasome activation in keratinocytes, providing important information during the *S. epidermidis*-keratinocyte interaction to activate the innate host defense (82).

### 3.3 Streptococcus spp

#### 3.3.1 Streptococcus agalactiae

*Streptococcus agalactiae* is an opportunistic β-hemolytic bacterium of the female genitourinary tract that can ascend during pregnancy, causing premature births and/or abortions. The high vaginal colonization of *S. agalactiae* is the main cause of vertical transmission, resulting in invasive infections such as pneumonia and neonatal meningitis (125). Understanding the pathogenesis of *S. agalactiae* will allow the development of effective vaccines and therapies against the pathogen (126). The secretion of IL-1β and IL-18 in human macrophages treated with β-hemolysin from *S. agalactiae* was associated with NLRP3 inflammasome activation during bacterial infection-mediated fetal death (127).

Several bacterial pathogens have evolved mechanisms to presentation sialic acid-binding immunoglobulin-like lectin ligands (Siglecs) on their cell surface to inhibit immune responses by molecular mimicry (83). *S. agalactiae* manipulates host immune cells through sialic acid mimicry, involving CD33-related inhibitory Siglecs to promote evasion of the host immune system. Siglecs are inhibitory receptors containing immunoreceptor tyrosine-based inhibition motifs in their intracellular signaling domains. Siglec-14 and -5 modulate the phagocytic and bactericidal responses of neutrophils and monocytes to *S. agalactiae*. The innate spectrum can also be modulated by the opposing mechanisms performed by Siglec-5, which inhibits inflammasome activation, and Siglec-14, which potentiates inflammasome activation in response to *S. agalactiae* infection, and may represent a therapeutic target against excessive inflammatory responses promoted by *S. agalactiae* (84). Additionally, vimentin has been described as a ligand of Siglec-14 after its secretion by activated macrophages, which may increase inflammasome activation (128). In addition, β protein induced binding to the Siglec-7 receptor, decreasing pro-IL-1β cleavage and IL-1β secretion, which may represent a mechanism to prevent innate immunity (129).

The induction of proinflammatory responses by protein kinase D (PKD) in *S. agalactiae*-mediated immune activation has been reported in murine and human macrophages (130). Protein kinase D is involved in cellular activities such as modulating cell signaling and vesicular traffic, transcription factor regulation, cellular detoxification, and inflammasome activation (85, 86). Study using placental macrophages infected by *S. agalactiae* showed the essential role of PKD during NLRP3 inflammasome formation/activation, inflammatory cytokine release, and NF-κB signaling processes (87). To date, the NLRP3 inflammasome has contributed significantly to host defenses, with β-hemolysin being the main stimulus of *S. agalactiae* to trigger a highly effective immune response through the NLRP3 inflammasome activation.

#### 3.3.2 Streptococcus pneumoniae

*Streptococcus pneumoniae* is a microorganism responsible for invasive diseases such as community-acquired pneumonia, sepsis, meningitis and otitis media, leading to high morbidity and mortality worldwide. However, the mechanisms used to activate the immune response remains to be clarified. Studies have shown NLRP3-dependent variation in IL-1β secretion by human cells infected with *S. pneumoniae*. Macrophages infected with serotypes that expressed low/non-hemolytic pneumolysin (PLY) release smaller amounts of IL-1β compared to macrophages infected with serotypes expressing active PLY (131). Additionally, the NLRP3 inflammasome was differentially activated by serotypes expressing PLY variants that triggered the production of IL-1 β and IL-18 cytokines (132). Hoegen and co-workers (133) showed the participation of PLY as an inducer of NLRP3 inflammasome and IL-1β expression in human THP-1 cells. In human neutrophils, PLY-mediated NLRP3 activation resulted in K+ efflux, caspase-1 activation, and pro-IL-1β cleavage (134). Furthermore, studies with murine neutrophils indicated that IL-1β activation was NLRP3 inflammasome-dependent, but AIM2 and NLRC4 inflammasomes-independent (135). In addition to NLRP3, PLY can also activate AIM2 inflammasomes (136).

Inactivation of NLRP3 or AIM2 inflammasomes increased mortality and bacterial colonization in the host, demonstrating the protective role of inflammasomes against *S. pneumoniae* infection (88). Moreover, IL-1β secretion was partially dependent on NLRP6 inflammasome activation during macrophage infection. Nlrp6−/− mice showed reduced mortality and bacterial colonization, as well as lower neutrophil and macrophage recruitment compared to wild-type mice, showing that NLRP6 plays a detrimental role in host defense against *S. pneumoniae* (89). Another study also demonstrated that Syk and JNK kinases were phosphorylated in a PLY-dependent manner during inflammasome activation by *S. pneumoniae* (90).

Receptor-interacting protein kinase 3 (RIPK3), a serine/threonine kinase, regulates both pro-inflammatory signaling for microbial elimination and excessive inflammation (137). Activation of the pulmonary NLRP3 inflammasome by *S. pneumoniae* was RIPK3-dependent. RIPK3 deficiency induced a decrease in NLRP3 expression, reduced IL-1β production, reduced bacterial clearance, with consequent severe lung damage and high mortality. RIPK3 also induced mitochondrial Ca^2+^ uptake, mitochondrial ROS production, and NLRP3 inflammasome activation *via* the AKT pathway against *S. pneumoniae* infection (138). In the lungs of infected mice, macrophage ATF3 activates the NLRP3 inflammasome to induce IL-1β secretion, which subsequently stimulates IL-17 secretion by γδ T cells. Interestingly, ATF3 (activating transcription factor-3) an important factor in the oxidative stress pathway of the endoplasmic reticulum promoted the activation and assembly of the NLRP3 inflammasome by ROS regulation during early *S. pneumoniae* infection. In addition, intracellular Ca^2+^ and ATP homeostasis, production of IL-1β and IL-23, and IL-17 secretion were also modulated by ATF3, demonstrating an important role in the survival of the host and in the elimination of *S. pneumoniae* (139).

Another important virulence factor, pneumococcal NanA, promoted extensive surface desialylation of infected cells and exaggerated inflammatory responses after infection. NanA induced the activation of multiple inflammatory pathways and cell death through the unregulated transfer of signals between TLR2 and siglec-5. NanA-mediated desialylation increased ASC oligomerization, caspase-1 activation and proteolytic cleavage of gasdermin D in infected THP-1 cells, suggesting that NanA inactivation may be a target to ameliorate inflammation and cytotoxicity caused by *S. pneumonia* (91).

Interestingly, biological aging favors a decrease in inflammasome activation during the inflammatory process caused by S*. pneumoniae*, showing that the elderly are more susceptible to *S. pneumoniae*. Elderly murine models demonstrated that misfolded proteins became increased with age, contributing to decreased assembly and activation of the NLRP3 inflammasome during *S. pneumoniae* infection (140).

#### 3.3.3 Streptococcus pyogenes

*Streptococcus pyogenes* is a pathogen responsible for injury and tissue destruction, bacteremia, multiple organ failure and death worldwide. Infections caused by *S. pyogenes* are highly contagious and can occur through hand contact with nasal secretions, airborne droplets, or surfaces contaminated with bacteria. Ineffective treatment of *S. pyogenes* infections can result in post-infection sequelae such as acute rheumatic fever and glomerulonephritis (141).

NLRP3 inflammasome signaling during *S. pyogenes* infection occurs through multiple mechanisms, depending on the cell line used, *S. pyogenes* M serotypes and *in vitro* or *in vivo* models. *S. pyogenes* LTA is a potent priming agent for the NLRP3 inflammasome in mouse bone marrow-derived macrophages, but not in differentiated human THP-1 cells (92). Isogenic mutant *S. pyogenes* revealed that streptolysin O (SLO) and streptolysin S were the main factors for inflammasome signaling in macrophages (142). SLO forms pores in host cell membranes, sufficient to release intracellular contents such as K^+^ and IL-1β (143). SLO also stimulates the activation of NLRP3 and caspase-1 in THP-1 macrophage-like cells (93). SLO-induced IL-1β-mediated inflammasome secretion required TLR and NF-κB-mediated cell signaling (94). In addition, a study demonstrated that partially active SLO, and therefore less cytotoxic, induced IL-1β secretion and delayed pyroptotic cell death. In this way, SLO-induced K+ efflux was able to activate the NLRP3 inflammasome (144). Moreover, macrophages were able to release IL-1β through P2X7 in response to *S. pyogenes* infection (145). Additionally, activation of caspase-1 by *S. pyogenes* required NF-κB, but was independent of P2X7R and TLR signaling (94).

Cysteine protease (SpeB) is able to cleave IL-1β contributing to alternative inflammasome-independent pathways. IL-1β signaling may protect the host but also lead to severe *S. pyogenes* infection (92). Cleavage of gasdermin A by the virulence factor SpeB was recently reported. This mechanism promoted a rapid burst of inflammation that limited invasive infection. Interestingly, SpeB directly cleaves gasdermin A, a family member expressed in keratinocytes, to release a pore-forming lytic subunit that can intercalate into membranes and lyse eukaryotic cells (95). These results demonstrated the important function of gasdermin A in defense of keratinocytes against the invasion of *S. pyogenes*, acting as a sensor of proteases of pathogenic bacteria.

#### 3.3.4 Streptococcus equi subsp. zooepidemicus

*Streptococcus equi* subsp. *zooepidemicus* (Sez) is an important pathogen responsible for infections such as meningitis, endocarditis and pneumonia in humans, acute and chronic endometritis in horses, and zoonosis (146). Studies have shown that microRNAs (miRNAs) play a crucial regulatory role in the human body. Regulation of the NLRP3 Inflammasome by Sez strains involved the downregulation of miR-223-3p in murine macrophages. Computational analyzes showed that miR-223-3p targets NLRP3 mRNA, whose overexpression of miR-223-3p suppressed NLRP3 inflammasome activation during Sez infection. Furthermore, miRNA-223-3p through regulation of the NLRP3/caspase-1 pathway caused inhibition of IL-1β and IL-18 secretion in response to the pathogen. Additionally, inhibition of miR-223-3p caused NLRP3 inflammasome hyperactivation. Consequently, miR-223-3p contributes to suppression of NLRP3 inflammasome activation and may be a therapeutic target for the treatment of Sez (96). Moreover, Gasdermin D-deficient mice were susceptible to intraperitoneal bacterial infection, with serious damage to the spleen and reduced secretion of cytokines IL-1β and IL-18 during *in vivo* infection (147). Furthermore, pore formation and pyroptosis in macrophages was gasdermin D-dependent, suggesting a defensive role of gasdermin D against Sez, providing a potential therapeutic target.

### 3.4 Cutibacterium acnes

*Cutibacterium acnes* (formerly called *Propionibacterium acnes*) plays a significant role in the development of acne, triggering immunologic defense that are vital for the pathogenesis and clinical appearance of acne vulgaris (148). In addition, *C. acnes* can also induce intervertebral disc degeneration (149). *C. acnes* produces proteases that participate in the inflammatory response of acne causing degradation of the extracellular matrix and proteolytic detachment of follicular keratinocytes. Activation of the NLRP3 inflammasome was dependent on metalloproteinase expression by *C. acnes* and ROS generation in sebocytes (97). *C. acnes* also induced ROS production, NLRP3 inflammasome activation and IL-8 release in HaCaT keratinocytes. Furthermore, the authors described the anti-inflammatory potential of the medicinal herb *Paris polyphylla* in reducing the inflammatory responses, proliferation and migration of keratinocytes caused by *C. acnes*, suggesting a new therapeutic for the treatment of acne (150). In addition, celastrol isolated from the *Celastraceae* family also exhibited anti-inflammatory activities by suppressing NLRP3 inflammasome activation promoted by *C. acnes* through inhibition of K63 deubiquitination of NLRP3, and providing evidence for its application in disease therapy (151).

Previously published data demonstrated increased activation of the NLRP3 inflammasome, caspase-1, caspase-5, IL-1β, IL-18 and gasdermin D after infection of *C. acnes*-infected nucleus pulposus tissue. In this study, it was also shown that the addition of the inflammasome inhibitor NLRP3 MCC950 and the thioredoxin binding protein (TXNIP) reduced the secretions of IL-1β and IL-18, implying an inflammatory response activated by *C. acnes via* the TXNIP-NLRP3 pathway. In this way, MCC950 can alleviate the inflammatory lesion and pyroptosis of *C. acne*s-infected cells, slowing the progression of disc degeneration, providing a new direction for the treatment of intervertebral disc degeneration (98).

The NLRP3 inflammasome plays a key role in acne lesions, raising interest in the suppression of this mechanism in the treatment of acne. Activation of NF-κB induced an increase in the expression of inflammatory factors during NLRP3 inflammasome activation. Thus, the suppression of the NF-κB pathway and the activation of the ERK1/2 MAPK signal transduction pathways caused inhibition of caspase-1 and NLRP3 expression, which could be considered therapeutic targets in the treatment of acne (99).

### 3.5 Corynebacterium pseudotuberculosis

*Corynebacterium pseudotuberculosis*, a facultative intracellular microorganism, belongs to the Corynebacterium-Mycobacterium-Nocardia-Rhodococcus group responsible for caseous lymphadenitis in goats and sheep. *C. pseudotuberculosis* is reported worldwide as the cause of significant economic losses affecting meat, wool and milk production, being difficult to detect in subclinically infected animals, to control and eradicate (152). *C. pseudotuberculosis* infections cause pyogranulomas and abscesses in organs and lymph nodes, with an increase in inflammatory cytokines. IL-1β is an important cytokine during the inflammatory phase in response to pathogens. Zhou and colleagues first described the activation of the NLRP3 inflammasome in IL-1β secretion in macrophages after infection by *C. pseudotuberculosis*. Activation of NLRP3 was independent of AIM2, but dependent on NF-κB, p38MAPK and partially dependent on TLR4 pathways. These results contribute to the knowledge of the mechanism of IL-1β secretion and of the host’s pro-inflammatory immune response in macrophages infected with *C. pseudotuberculosis* (100).

### 3.6 Bacillus anthracis

*Bacillus anthracis*, spore-forming Gram-positive bacteria, efficiently kills infected hosts through the systemic action of secreted edema toxin (EdTx) and lethal toxin (LeTx) (153). LeTx targets polymorphonuclear and mononuclear cells during the early stages of infection, paralyzing the host’s innate immune defenses and allowing bacteria to spread and cause systemic infection (154). Furthermore, direct or direct activation of caspase-1 by the NLRP1B inflammasome in response to LeTx was related to macrophage killing (101). Recent publication has shown the involvement of TNFR1, TNFR2 receptors, and the MAPKK/p38/MK2 signaling pathway in LeTx-intoxicated macrophages during NLRP3 inflammasome activation dependent on RIPK1 activity (155).

In contrast to activation of the NLRP3 sensor, little is known about the requirements for NLRP1B activation. Cell death was induced by LeTx toxin and mediated by the NLRP1B inflammasome and proteasome activity. However, inhibition of components such as caspase-1, K^+^ channel, cathepsin B and heat shock can block this activity. In this way, proteasome inhibition could delay the time of death caused by the LeTx toxin (102). When IL-1β and IL-18 are present, the LeTx toxin activates NLRP1B in toxin-responsive rodent macrophages, while resistant macrophages only suffer the consequences of cleavage of LeTx’s mitogen-activated protein kinase and others subtracts. Importantly, LeTx was not able to regulate or activate IL-1β release or induce the formation of murine neutrophil NETs. LeTx also did not induce neutrophil pyroptosis, despite cleaving cytosolic mitogen-activated protein kinase substrates, suggesting a complex mechanism of interaction between different cell types and mediators *in vivo* that differs from *in vitro* or *ex vivo* models (156). Therefore, it is important to know the inflammasome activation in a multicellular context of inflammatory response to a bacterial toxin.

### 3.7 Clostridioides difficile

*Clostridioides difficile* is the leading cause of hospital-acquired infection, resulting in pseudomembranous colitis, toxic megacolon, and even death. The diagnosis of high inflammatory level is a predictor of severe disease with high morbidity and mortality. Toxins A and B produced by *C. difficile* can stimulate a potent pro-inflammatory response with activation of IL-1β, IL-6, IL-8 and TNFα with inflammasome activation. *C. difficile* toxins A/B also synergize with MyD88 in the production of IL-23. Furthermore, inhibition of Rho protein GTPase activity by toxins A/B induced the cleavage of pro-IL-1β and IL-8, resulting in the recruitment of innate immune cells (157).

The pyrine inflammasome does not directly recognize pathogens or host-derived molecules, but responds to disturbances in cytoplasmic homeostasis caused by infections leading to inactivation of RhoA GTPase. Activation of these signaling pathways increases IL-1β production and promotes IL-1 receptor expression with uncontrolled activation of the pyrin inflammasome in neutrophils (158). Furthermore, pyrin inflammasome activation can be affected by RIPK3, which modulates the rapamycin (mTOR) pathway, regulating the expression of the gene encoding pyrin and pyrin inflammasome activation (103).

Interactions between bacterial virulence factors and cell receptors are fundamental as possible targets for treating bacterial infectious diseases. Surface layer proteins of *C. difficile* (SLPs) bind to the lipid rafts, induced caspase-1, and IL-1β production in dose-dependent manners (159). The production of IL-1β induced by *C. difficile* in macrophages was dependent on caspase-1, MyD88 and TLR2, considered critical in the production of pro-IL-1β. Additionally, the toxigenic *C. difficile* in the cytosol was necessary for inflammasome activation through the ATP-P2X7 pathway with consequent loss of membrane integrity, release of intracellular content and caspase-1-dependent cell death. In addition, SLPs were released from pyroptotic cells. Consequently, MyD88 and TLR2 were essential components in pro-IL-1β cleavage and inflammasome activation *via* ATP-P2X7, responsible for inflammation-mediated bacterial clearance during *C. difficile* infection (104). Together, these results contribute to the identification and understanding of the essential factors of infection caused by *C. difficile*, allowing new therapeutic strategies to prevent the disease.

### 3.8 Clostridium spp

Recently, evidence has demonstrated the involvement of mixed-lineage protein kinase (MLKL) in mediating the immune response against severe gas gangrene and enterocolitis caused by *Clostridium perfringens*. MLKL plays an important role in innate immunity during the process of necroptosis. Regarding *C. perfringens*, MLKL promoted bacterial eradication, increased host survival and resolution of inflammation, demonstrating that MLKL-NLRP3 inflammasome confers protection to invading pathogens (160).

Another species, *Clostridium septicum*, a pathogen that causes sepsis and gas gangrene, was able to activate the inflammasome complex in mice and humans. *C. septicum* secretes a α-toxin responsible for binding to glycosylphosphatidylinositol proteins anchored in the plasma membrane of host cells, forming pores that allow the efflux of Mg^2+^ and K^+^ ions. The efflux of these cytosolic ions triggers NLRP3 inflammasome activation, caspase-1 and gasdermin D activation, IL-1β and IL-18 secretion. The innate detection of the α-toxin is essential for the recognition of *C. septicum* infection, whose therapeutic blockade may be able to prevent sepsis and death caused by microbial toxins (105).

## 4 Cross-regulation of inflammasome and autophagy activation by Gram-positive bacteria

The induction of autophagy acts as the first line of defense during infection by intracellular pathogens, including Gram-positive bacteria (161). Autophagy is a mechanism that acts on cellular homeostasis, allowing the generation of amino acids, recycling and elimination of non-functional organelles (162, 163). Thus, autophagy is upregulated in response to the need for amino acids and damaged organelles, including mitochondria (mitophagy).

The connection between autophagy and inflammasome activation was first reported in 2008. Autophagy dysfunction can lead to hyperinflammation and excessive activation of the NLRP3 inflammasome, acting as an inflammasome regulator. Elimination by autophagy of NLRP3 inflammasome components reduced inflammasome activation and inflammatory response. On the other hand, the inflammasome can also regulate the autophagic process necessary for the balance of the inflammatory response, preventing excessive and harmful inflammation (164). Autophagy has a protective role in some NLRP3 inflammasome-associated inflammatory diseases, including sepsis caused by many Gram-positive bacteria (165). CD46 is a glycoprotein expressed by human cells that binds to pathogens such as *S. pyogenes*. Mechanisms employed for intracellular *S. pyogenes* autophagy include the binding of the CD46 receptor with bacterial M Protein. After CD46 activation, *S. pyogenes* is targeted to autophagosomes. CD46 can control early infection of the pathogen by autophagy, as shown that the CD46-Cyt-1/GOPC pathway participated in directing *S. pyogenes* to autophagic degradation. This pathway induces the formation of autophagosomes that efficiently sequester and degrade pathogens, optimizing the innate cellular defense against pathogenic microorganisms (166).

Pathogens in the cytosol are ubiquitinated and captured by LC3-positive double membranes *via* autophagy receptors (167). Interestingly, SLO *S. pyogenes* promoted escape from phagosomes, ubiquitination and recognition by the p62, NDP52 and NBR1 autophagic adapters. Furthermore, studies have shown that the secreted protein, NAD-glycohydrolase (Nga), is translocated to the host cytosol *via* SLO, promoting inhibition of LC3 formation and suppression of phosphatidylinositol 3-kinase catalytic subunit type 3 (PIK3C3)-dependent autophagosome formation during early infection, facilitating intracellular proliferation of *S. pyogenes* (168). The p62, NDP52 and NBR1 adapters are critical for the recognition of ubiquitinated *S. pyogenes* and recruitment of LC3 before autophagic degradation. Moreover, expression of SpeB *S. pyogenes* protected the bacterium from autophagy through p62, NDP52 and NBR1 degradation (169). Interestingly, *L. monocytogenes* suppressed LC3-associated phagocytosis through modulation of mitochondrial Ca^2+^ signaling to survive in the intracellular environment. Macrophage invasion by *L. monocytogenes* induced mitochondrial Ca^2+^ uptake through the mitochondrial Ca^2+^ uniporter, promoting increased production of acetyl-coenzyme A by pyruvate dehydrogenase, decreasing the formation of LC3-associated phagocytosis. Consequently, modulation of mitochondrial Ca^2+^ signaling increased *Listeria* survival in the cytosol, demonstrating the important role of mitochondrial metabolism in *Listeria*-host cell interactions (170). Furthermore, LLO forms pores in the phagosomal membrane immediately after uptake by bacteria, and *L. monocytogenes* is able to escape autophagic degradation (171). The virulence factor, ActA, from *L. monocytogenes* is implicated in intracellular motility and evasion of autophagy. Despite contradictory results with Δ*actA* mutants, results with HeLa cells showed a time-dependent increase in colocalization with LC3, which may represent an important escape mechanism from ActA-dependent autophagy (172). Moreover, the synergistic effects of ActA and *L. monocytogenes* phospholipases (PlcA and B) for escape from autophagy was reported. *PlcA* and *plcB* mutants were rapidly targeted to autophagosomes compared to wild-type bacteria (173).

Previous studies demonstrated that blocking autophagy potentiated inflammasome activity, while stimulation of autophagy promoted inflammasome ubiquitination with the recruitment of the p62 autophagic adapter, which aided in autophagosome targeting. In this way, autophagy accompanied inflammasome activation to moderate inflammation (174). Studies have also shown that impaired mitophagy increases activation of NLRP3, while induction of mitophagy reduces activation of NLRP3 (175). Supporting this notion, a study by Shi and co-workers (174) showed colocalization of the NLRP3 inflammasome with autophagosomes and that inhibition of the autophagic pathway by post-translational modifications of NLRP3 caused increased activation of the NLRP3 inflammasome, suggesting a negative feedback to prevent the excessive inflammation. Furthermore, induction of autophagy increased IL-1β secretion in response to NLRP3 inflammasome activation in macrophages (176). The regulation of IL-1β release by the autophagic machinery is complex and requires further investigation, and may be influenced by different inflammasome activators, autophagy inducer/inhibitor and cell lineage types.

Interestingly, experiments performing silencing of NLRP3 or ASC in microglia suppressed caspase-1 activation and increased autophagy. In addition, inhibition of autophagy by the NLRP3 inflammasome occurred through cleavage of the TRIF signaling molecule by caspase-1 (177). Inflammasome activation promoted a rapid blockade of caspase-1 dependent mitophagy, resulting in the accumulation of mitochondrial DNA and dysfunctional mitochondria. The increase in damaged mitochondria produced the increase in mitochondrial ROS allowing greater activation of the inflammasome, amplification of the inflammatory response and pyroptosis in macrophages (178).

**Figure 2:**
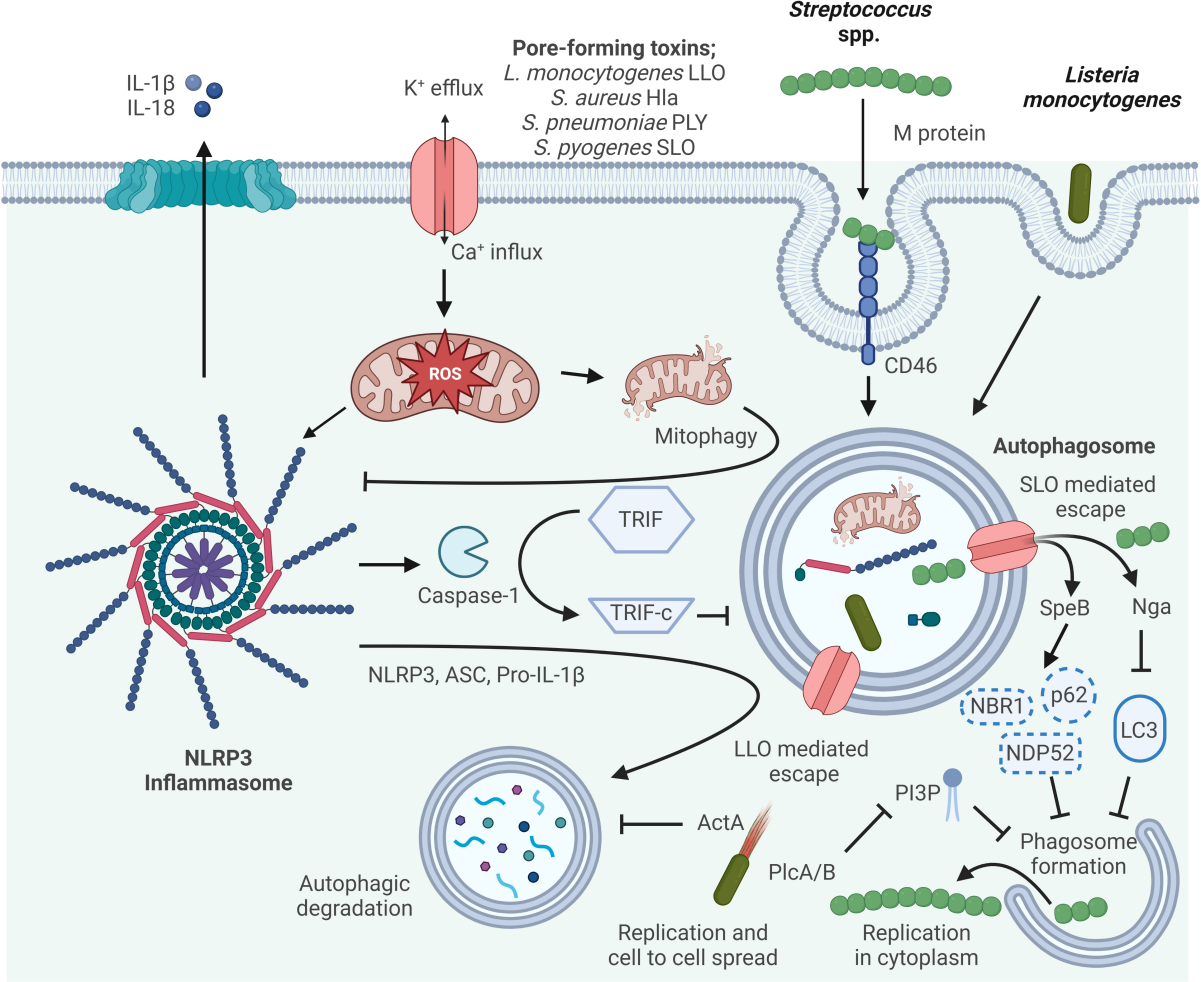
Mechanisms of autophagy/NLRP3 inflammasome by Gram-positive bacteria interaction. Autophagy can inhibit NLRP3 inflammasome activation by reducing ASC, increasing NLRP3 phosphorylation, and eliminating ROS. Activated Caspase-1 directly cleaves TRIF, thereby diminishing TRIF-mediated autophagy. The M protein of *S. pyogenes* binds the CD46 receptor to facilitate internalization, and subsequently *S. pyogenes* escapes from the phagosome *via* streptolysin O (SLO)-dependent pore formation. In the host cytosol, streptococcal pyrogenic exotoxin B (SpeB) degrades the autophagy adapter proteins p62, NDP52 and NBR1, and NAD-glycohydrolase (Nga) disrupts LC3 formation thereby inhibiting phagosome formation and escape from autophagy allowing for *S. pyogenes* cytosolic replication. LLO, listeriolysin O, pore-forming acts to damage and disrupt the vacuole membrane. Surface protein A (ActA) recruits the host Arp2/3 complex and Ena/VASP to the bacterial surface, which disguises the bacteria from autophagic recognition. Phosphatidylinositol-specific phospholipase C (Plc) A and B expressed by *L. monocytogenes* interfere with the production of phosphatidylinositol 3-phosphate (PI3P), a phospholipid required for phagosome formation. Escape from autophagy allows for *L. monocytogenes* replicating within the host cytosol and cell-to-cell spread.

NLRP3 autophagy-inflammasome regulation is very complex and the influence is bidirectional (**Figure 2**). However, the relationship between autophagy and the NLRP3 inflammasome should be further explored. For example, an in-depth analysis to understand the physiological, pathological and inflammatory processes of autophagy relevant to the future development of drugs to treat inflammatory diseases.

## 5 Concluding remarks and future perspectives

Recent work has expanded our understanding of inflammasome activation and how Gram-positive bacteria can activate, evade, and manipulate the inflammasome and the downstream mediated immune responses. The success or failure of a pathogen is dependent on its ability to adapt to a new host environment. The host immune system on the other hand has evolved to effectively defend against the pathogens attack. This attack-defense strategy has been the driving force for both the host and the pathogen to develop new defenses to cope with new attack mechanisms. For example, Gram-positive bacteria insert pore-forming toxins (PFTs) into the cell membrane to facilitate the infection cycle. Some PFTs are also involved in disruption of the phagosome, thereby gaining cytosolic access, which is critical for their virulence strategy and lifestyle. However, changes in cytosolic potassium concentrations as a consequence of pore formation, or release of PAMPs from a ruptured phagosome, leads to activation of inflammasomes. This continuous host-pathogen interaction has put selective pressure on the pathogen to make changes to their virulence factors to try to inhibit immune responses or to avoid immune recognition. Similarly, inflammasomes have evolved to respond not only directly by binding to microbial ligands, but also indirectly by sensing common infection strategies employed by a variety of pathogens. Understanding this constant battle and the underlying mechanisms by which Gram-positive bacteria modulate host innate immune responses is essential for future therapeutic development.

## Author contributions

AMK-G and PN wrote and revised the manuscript. All authors contributed to the article and approved the submitted version.
